# Unusual Site Of Permanent Pacing: A Case Report

**Published:** 2009-03-15

**Authors:** Rakesh Yadav, Sharad Chandra, Nitish Naik, CM Nagesh, SS Kothari

**Affiliations:** Department Of Cardiology, Cardiothoracic Sciences Centre, All India Institute of Medical Sciences, Ansari Nagar, New Delhi - 110029, India

**Keywords:** subclavian, pacemaker, iliofemoral, transiliac

## Abstract

Subclavian route is a standard way of performing a permanent pacemaker. However in cases with superior vena caval or bilateral subclavian occlusion and bilateral infection of pacemaker site, alternative site is warranted. Epicardial route needs general anesthesia and has its own problems. Iliofemoral route has been used previously but has more lead related problems and patient discomfort. Here we are reporting a case in which transiliac pacing was done due to both right and left pacemaker site active infection and to avoid the chance of lead dislodgement, an alpha loop was made in the right atrium.

## Case Report

**Figure 1:**
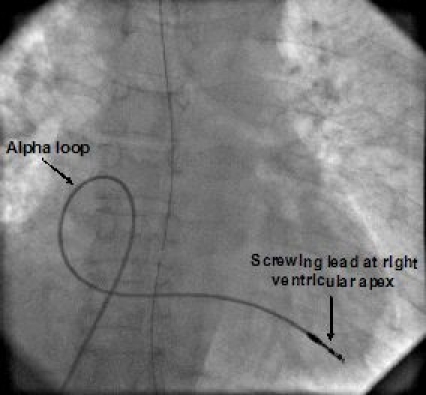
Fluoroscopic image in anterioposterior view showing screwed pacing lead at right ventricular apex with an alpha loop in right atrium

 A 50-year female presented to our institute with both right and left pacemaker site infection and protrusion of pacemaker from the left insertion site. This patient presented to a local physician 4 months back with history of syncopes and was found to have complete heart block. Single chamber ventricular pacemaker was implanted from the right subclavian approach. The pacemaker got infected so it was explanted and a new pacemaker was inserted from the left subclavian route in the same hospital. After 15 days, that too got infected. The patient was given a trial of antibiotics for 3 months but infection could not be controlled. Then the patient was referred to our institute. At the time of presentation the right pocket was still infected and discharging sinus was formed. The pacemaker was extruded and active discharge was coming out from left site. Patient was on oral antibiotic and there were no systemic signs of septicemia. Patient was started on intravenous antibiotics and after 3 days, pacemaker was explanted. Both wounds were debrided and both leads were extracted. Patient was put on temporary pacing through right transfemoral route. Intravenous antibiotics were given empirically as cultures were sterile. After 5 days of antibiotics treatment, patient was taken for permanent pacemaker implantation from the right iliac route as both anterior chest walls were infected.  In supine position, the incision was made 1 inch above the inguinal ligament. Dissection was done and external iliac vein was dissected extraperitonealy taking extreme precaution of not to expose peritoneum. The temporary lead which was put from the right femoral vein was used to locate the iliac vein. Then puncture of external iliac was done using 16-gauge needle and a wire was inserted, over which peel off sheath (DAIG-SJM) was introduced. A long 85 cm screw in lead (ISOFLEX 1688T) was inserted and screwed at right ventricular apex. An alpha loop was made in the right atrium so that the lead can not be stretched by its own weight avoiding lead displacement (Figure 1).

**Figure 2:**
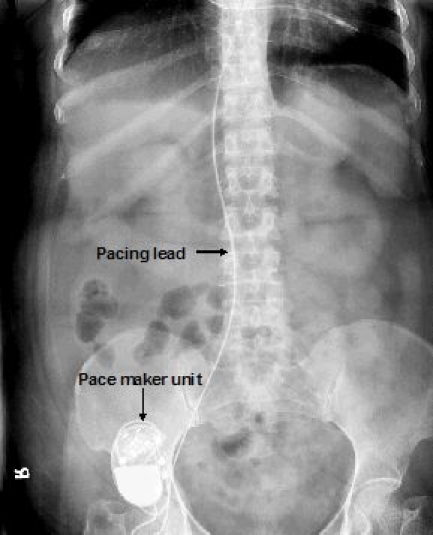
X-ray in anterioposterior view showing pacemaker unit lying under rectus sheath with pacing lead going through external iliac vein to inferior vena cava. Pacemaker unit is looking lateral and down  because of patulous abdomen and X ray has been taken in erect posture.

 A second incision was given lateral to umbilicus over rectus sheath for putting pacemaker unit. The lead was then tunneled subcutaneously and connected to the pacemaker (VVIR, Regency 2400L) which was put from the second incision (Figure 2).

Both wounds were closed. Patient was immobilized for a day and discharged on 7th day. After 12 months follow up patient was fine with good pacemaker performance as assessed by Holter and repeated pacemaker interrogation (Pacing threshold 0.8V, resistance 630Ω and R wave amplitude 7 mV).

## Discussion

 Trans subclavian route is a standard way of putting permanent pacing leads. In cases of inaccessible subclavian route because of blocked superior vena cava, occlusion of both subclavian veins, pacemaker site infection of both right and left side and previous epicardial lead failure, alternate route is warranted [1,2]. Epicardial route needs general anesthesia and has its own problems [3]. Transfemoral route has been accepted as an alternative site especially in children [4]. In India, persons squat more because of many religious reasons and social customs prevailing in the society, so transfemoral lead placement can cause many problems like patient discomfort and lead fracture because of repeated bending of the lead due to squatting. Transiliac route should be a better option in this situation as squatting will not cause lead damage and discomfort to the patients as lead is totally above the inguinal ligament. Transiliac technique takes almost same time as subclavian with no chance of occurrence of peneumothorax. Major problem with this approach is lead dislodgement. Ellestad and French reported 11-21% rate of atrial lead dislodgement and 5-7% rate of ventricular lead dislodgement with iliac vein approach [5]. This dislodgment can be taken care of by screw-in type lead, alpha loop in right atrium and by putting only ventricular lead as we did in this case. Incidence of asymptomatic deep vein thrombosis is up to 30% in transfemoral pacing similar to asymptomatic subclavian vein thrombosis in subclavian approach [6]. However in our patient, there is no evidence of deep vein thrombosis on venous Doppler after 6 months. Complications as lead fracture, thrombophlebitis, thromboembolism and infection can occur in transiliac pacing but it is not known whether rates of these complications are equal or higher than those of superior vena cava approach [7]. A case report of dual chamber ICD implantation through left iliac vein was described from our own  country. In that case, subcutaneous pocket was created in left subcostal region so that this site could be used as an electrically active electrode for defibrillation but the technique of implantation and importance of extraperitoneal route and alpha loop  were not described [8]. Transiliac route requires help of surgeon for the dissection of external iliac vein and extraperitoneal route should be strictly followed. The alpha loop which we made in the right atrium also seems to be important because it will avoid traction in the lead during standing, decreasing the risk of lead dislodgement.
